# 

**Published:** 1872-05

**Authors:** 


### Contents of May Number.

### ORIGINAL DEPARTMENT.

ART. I.—Clinical Experiences in private practice. Complicated chronic case. By Z. C. McElroy, M. D. Zanesville, Ohio.................. 365
ART. II.—A case of Placenta Prævia, terminating in death on the thirteenth day after delivery. By F. W. Hutchinson, M. D. Minneapolis, Minn.................................................. 373
ART. III.—On the recent advances in the Theory of Vision. By H. Helmholtz. Translated by F. W. Abbott, M. D........................... 376

### MISCELLANEOUS.

Report of Two Cases of Inversion of the Uterus, with Remarks and a Description of the Uterine Repositor. By Prof. James P. White, M. D.......................................................... 383
A new method of applying steam to the middle ear and of inhaling medicated vapor with a new Steam Atomizer........................... 396

### EDITORIAL DEPARTMENT.

Obituary.—Dr. Chas. A. Weston, Nicolaus, Cal......................... 398
American Medical Association............................................ 398
Books Reviewed:
Can Chloroform be used to facilitate Robbery? By Stephen Rogers, M. D.............................................. 399
Transactions of the American Ophthalmological Society..... 399
Eating and Drinking. By Geo. M. Beard, M. D............ 399
Stimulants and Narcotics. By Geo. M. Beard, M. D........ 399
Plain Talk about Insanity. By T. W. Fisher, M. D.. ..... 400
Animal and Vegetable Parasites of the Human Skin and Hair. By B. Joy Jeffries, A. M., M. D...................... 400
An Introduction to Pathology and Morbid Anatomy. By T. Henry Green, M. D........................................ 400
Pulmonary Consumption. By C. B. J. Williams, M. D., F. R. S., & Chas. T. Williams, M. A., M. D........ .... 401
Injuries to Nerves and their consequences. By S. Weir Mitchell, M. D............................................. 401
The Atlantic Monthly.................................................... 402
Books and Pamphlets Received............................................ 402
Toner's Medical Register and Directory.................................. 404

